# Life Insurance and Sanitary Science

**Published:** 1911-08

**Authors:** 


### Abstracts and Selections.

### Life Insurance and Sanitary Science.

The following address was delivered before the Louisiana State Medical Society by Dr. J. H. Florence, medical director of the Great Southern Life Insurance Company, Houston, Texas, recently:

It is a well known fact that a large proportion of the profit made by life insurance companies arises from a saving in mortality; hence, the fewer the number of deaths among policyholders the more profitable the business. Therefore, it requires no thought to deduce that when the sanitary surroundings in any locality are good, and every possible agent conducive to good health and long life is used, the life insurance in that locality will be conducted upon a profitable basis. The reverse also holds. Where business is put on the books from unhealthful and unsanitary localities, with all the surroundings detrimental to good health, it only remains to be said that such business is carried at a loss, or at a minimum profit.

The question naturally arises: Why so much concern about the welfare of the stockholders? We may rest assured that the premiums on all policies are loaded to cover lives in unhealthful territory, also to cover those shortened by the non-observance of the laws of health. This loading is necessary to protect the companies against loss arising from unsanitary surroundings, which would not occur were the policyholders residing in localities conducive to good health. In other words, a citizenship which permits preventable diseases and unsanitary conditions to exist, and death to run rampant, must "pay the freight," not only with the life blood of its individual members, but in loss of time and money. Moral: The nearer ideal the sanitary conditions and the nearer perfect the hygienic surroundings the lower will be the death rate maintained, while the losses of life insurance companies will be minimized. There is no doubt that as long as these filthy conditions exist, and as long as preventable diseases go unbridled, just so long will an insurance public pay for its negligence. I say negligence because neither municipal or health officers will do very much along sanitary lines unless there exists a popular demand for action.

Educational work must begin with the people. The sanitary officers, in most cases, already have the education, brains and good judgment to inaugurate and enforce crusades for health betterment—they only need the indorsement and demand of the public.

It does seem that could the insuring public fully realize the excessive amount it pays for life insurance, by reason of existing conditions, there would be a general and permanent move made to eradicate all those nuisances which are known to create disease and death, thereby materially lowering the mortality, thus enabling the life insurance companies to make cheaper rates, also saving time and money now expended on account of sickness. Surely the time for an awakening is at hand, which should be welcomed and encouraged by influence, brain and resources.

From a cold business point of view, it is not profitable to life insurance companies to collect an excess for deaths that ought not to occur, but do and will as long as the present conditions exist, as it is inevitable that it must be paid out. The longer a policyholder lives the more he pays; also the fewer the deaths the greater the profit to the companies; so it can readily be reckoned that the companies are eager for this much needed reform.

It is a deplorable fact that we pay a heavy penalty for the privilege of living and dying in unhealthful surroundings and ignoring all the laws of health. This I know is a severe arraignment of the intelligence of our people, nevertheless, it is true. Let an agent of a fire insurance company inform you that your premium rate will double if you store explosives in your home, or install defective electric wiring, and you waste no time in removing the explosive or correcting the wiring. Why should we not be as alert to our physical and financial interests when so notified by those well-informed and drilled in laws and measures conducive to good health and long life?

Two large companies in the North, growing impatient at the delay of the people and authorities in adjusting these matters, have begun a system of education by mail among their policyholders. Some companies and fraternal orders have enployed nurses to visit the homes of the policyholders advising them along hygienic lines; they have also established sanitariums for the benefit of those becoming sick after securing policies. These methods have their limitations, but it is a move in the right direction. It is a notable fact that a few companies refuse to enter our Southern States; others enter but restrict their operations to certain districts. Evidently there is a reason, and I am constrained to believe that it is not an imaginary one, but is based upon mortality experience tables. I am not unmindful, however, that there has been an improvement in these districts since the mortality tables in question were compiled. Surely our Southland has borne this stigma long enough.

When sanitation and hygiene are taught daily in our schools, when sane health laws are passed and consistently enforced, and when the vast financial savings of such procedure dawns upon the average man, we may expect an awakening—but not till then. It is a well known fact that when you prove to the average citizen that his pocketbook suffers by any neglect upon the part of himself, or his State, there is found an early remedy; and if we wish to accomplish much we must make that the basis of our plea. No better proof of this assertion can be found than by drawing a parallel between the amount of money appropriated and expended by our lawmakers for the protection of live stock and agricultural products and the sum devoted to the protection of the health of the people. We can not believe that they deliberately place the hog above the baby, the sheep above the voter, or the cotton plant above the mother, but must conclude, in all charity, that their attention has never been called to this inconsistency.

Every one who reads the daily papers and current literature has found in almost every issue during the past year interesting information concerning the conservation of our natural resources, forests, minerals, etc. It was easy for our lawmakers to see why the hand of greed should be stayed and our forests and minerals conserved, and appropriations were made for same. How much more important is the conservation of the health of our people; yet we see this set aside for the most trivial legislation. While we give due credit to those philanthropists who are donating from their large private means to the conservation of health, we bow our heads in humiliation that such a course is necessary while we enjoy such a great and much boasted civilization.

We have temporized with sentiment and moral suasion long enough. Let us be "up and doing." Let the unselfish medical profession, with its untiring energy, not only keep up present meritorious tactics, but get immediately at the heart of matters by busying ourselves with those who make and execute our laws as well as with the individual citizen, making our demands upon an economic basis—a saving of dollars and cents to the commonwealth, and ere long we will hear the rumblings of a popular demand that will reach the desired end. Then our profession will come into its own and reap the reward that a diligent, scientific and sacrificing promoter of good should receive.

One of the greatest factors in bringing about this Utopia must be the organized medical bodies; they should by precept and example, in season and out, educate the people, give publicity to these needed reforms, deliver public addresses, and give their expressions to the secular press in no uncertain terms.

The press is a powerful agent, and it has at all times been willing to open its columns for the dissemination of scientific knowledge for the general betterment of health. The people look to the medical profession (and rightly so) for their knowledge and guidance, and should not be disappointed. Without correct information, sane legislation and wise administration of sanitary and hygienic laws the people are practically helpless.

It is to be hoped that the medical profession, as a united body, will not hesitate longer to grasp the situation, but will take immediate action, to the end that happiness, health and prosperity may prevail, disease be robbed of its horrors, life extended and death be cheated of its untimely prey.—*Houston Post.*

---

For Rectal Fissures.—A 1 per cent solution of atropin will deaden sensation in a fissure. Oil enemata will make defecation less painful. A good suppository is the following:

| | | |
|---|---|---|
| ℞ | Bismuthi subnit | 6.0 grams. |
| | Resorcinol | 0.5 grams. |
| | Balsam Peruv | 1.5 grams. |
| | Ol. theobrom | 19.0 grams. |
| | Ung. cerat | 2.5 grams. |

M. f. suppos, no. XII.

—*Therapeutic Medicine.*

